# The Value of Intraoperative Neurophysiological Monitoring During Patient Positioning in Spine Surgery: A Preventive Strategy

**DOI:** 10.7759/cureus.73662

**Published:** 2024-11-14

**Authors:** Paula Rebelo, Cristina P Sousa, Manuel T Campos

**Affiliations:** 1 Anesthesiology Department, Unidade Local de Saúde de Matosinhos, Matosinhos, PRT; 2 Anesthesiology Department, Centro Hospitalar Trás-os-Montes e Alto Douro, Vila Real, PRT; 3 Neuroanesthesia, Unidade Local de Saúde de Santo António, Porto, PRT

**Keywords:** anesthetic management, cervical myelopathy, intraoperative neurophysiological monitoring, patient positioning, spinal cord injury

## Abstract

Cervical spine surgery in patients with myelopathy poses a substantial anesthetic challenge, primarily due to the risk of secondary spinal cord injury (SCI). Traditionally, concerns have centered around cervical movements during intubation. However, limited evidence supports a direct link between intubation and SCI, so anesthesiologists must consider other factors, including patient positioning, spinal perfusion pressure, and direct surgical complications. In this context, multimodal intraoperative neurophysiological monitoring, including somatosensory (SSEPs) and motor evoked potentials (MEPs), is important for real-time assessment of spinal cord integrity.

We present a 73-year-old male with cervical spondylotic myelopathy following a fall. The patient exhibited left-sided hemiparesis and sensory deficits at the C6 level. Imaging revealed significant C4-C5 and C5-C6 spinal cord deformation, leading to the decision for anterior cervical discectomy and fusion. Given the risk of SCI, anesthesia was managed with multimodal neurophysiological monitoring, including bilateral processed electroencephalogram, bilateral cerebral oximetry via near-infrared spectroscopy (NIRS), and nociception monitoring (ANI®). Awake fiberoptic intubation was performed under sedation to minimize cervical movement. Anesthesia was maintained with propofol and remifentanil infusions, without muscle relaxants. Neurophysiological monitoring, including SSEPs and MEPs, began before positioning to establish baseline neural function. The patient was positioned for surgery without significant changes in evoked potentials. A transient hypotensive episode post-intubation was corrected with ephedrine. The surgery proceeded uneventfully, and the patient awoke with no additional neurological deficits. At three-month follow-up, he had recovered normal muscle strength.

Cervical myelopathy increases the risk of SCI due to the cord's heightened sensitivity to minor insults. Recognizing this risk foresees the need for heightened vigilance and advanced intraoperative neurophysiological monitoring to prevent the exacerbation of pre-existing lesions and mitigate the risk of secondary injury from positioning and controlled hypotension. This case highlights the necessity of broadening anesthetic vigilance beyond intubation to include patient positioning and spinal perfusion management. Multimodal intraoperative neurophysiological monitoring, initiated before critical phases such as patient positioning, is vital in the management of cervical spine surgeries in patients with myelopathy. This proactive approach minimizes the risk of secondary spinal cord injury and improves postoperative outcomes by enabling early detection of neural compromise and timely adjustments during surgery.

## Introduction

Patients with myelopathy undergoing cervical spine surgery present a significant anesthetic challenge. Traditionally, the focus has been on airway management during intubation, given the potential for cervical movements to cause neurological deterioration. However, despite long-standing concerns, there is limited evidence directly linking cervical movements during intubation to secondary spinal cord injury (SCI) [1]. Thus, while vigilance during intubation remains essential, anesthesiologists must broaden their focus to include other potentially overlooked variables.

One such variable is patient positioning during surgery. Achieving optimal surgical exposure often necessitates placing the patient in positions, such as neck hyperextension, that could increase the risk of SCI [2]. Unlike the brief duration of cervical movements during intubation, surgical positioning can last for several hours, potentially exerting a more significant impact on the spinal cord. The ideal patient positioning is therefore critical for procedural success and minimizing the risk of secondary injury [3].

Furthermore, maintaining adequate spinal perfusion pressure is important to prevent ischemic complications during anesthesia, particularly in susceptible patients with pre-existing spinal cord pathology. Additionally, direct surgical complications, though often underestimated, can lead to SCI if not promptly identified and managed.

In this context, multimodal intraoperative neurophysiological monitoring, including somatosensory and motor-evoked potentials (SSEPs and MEPs), emerges as an invaluable tool. It allows for real-time assessment of neural integrity, providing early warning signs of potential injury. This monitoring is not only essential for detecting the effects of surgical manipulation but also for evaluating the impact of anesthetic and positioning strategies on spinal cord function [4].

This case report describes the successful anesthetic management of a patient with cervical myelopathy in a traumatic context undergoing anterior cervical discectomy. It highlights the importance of a comprehensive, multimodal approach that goes beyond airway management to include vigilant monitoring of positioning, spinal perfusion pressure, and potential surgical complications, ultimately aiming to prevent secondary spinal cord injury and improve patient outcomes.

## Case presentation

**Figure 1:**
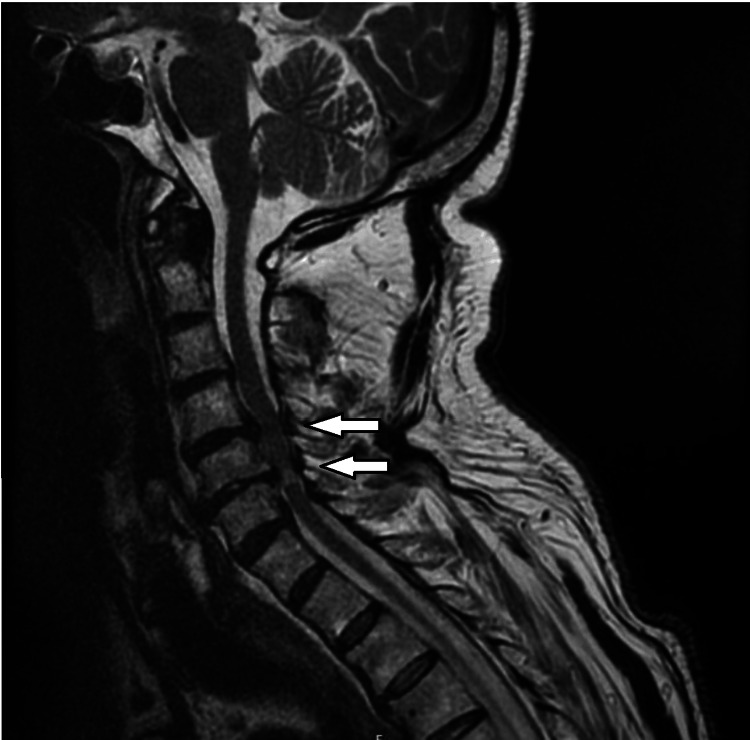
Cervical MRI Arrows: medullary narrowing at C4-C5 and cervical spondylotic myelopathy centered behind the C5-C6 disc

A 73-year-old, 84 kg, 175 cm (body mass index: 27.4 kg/m2), American Society of Anesthesiologists (ASA) physical status III male was admitted to the emergency department following a fall from standing height, which resulted in acute neurological deficits, including left hemiparesis (degree 4/5 in the left lower limb; degree 4/5 and 2/5 in the left upper limb, proximally and distally, respectively), hyporeflexia, slightly extensor plantar reflex, and positive Hoffman test with a sensory deficit level at C6 bilaterally. Cervical computed tomography (CT) revealed discovertebral degenerative alterations at C4-C5 and C5-C6, causing significant deformation of the spinal cord, particularly at C5-C6. There was also foraminal stenosis, more pronounced at C5-C6. These findings were confirmed by cervical magnetic resonance imaging (MRI), which showed cervical spondylotic myelopathy centered behind the C5-C6 disc and relevant medullary narrowing at C4-C5, as seen in Figure 1.

The patient was consequently referred for anterior cervical discectomy and fusion at C4-C5 and C5-C6 and maintained a cervical collar until surgery.

The patient's past medical history included cardiovascular risk factors (such as arterial hypertension, dyslipidemia, type II diabetes mellitus, and overweight), Hashimoto’s thyroiditis, and dilated cardiomyopathy with moderate ejection fraction depression. Preoperative exams revealed no further significant findings.

In addition to standard ASA monitoring, the patient was monitored with invasive blood pressure monitoring, a bilateral processed electroencephalogram with BIS^TM^ to access the hypnotic state, bilateral cerebral oximetry (rSO2) via near-infrared spectroscopy (NIRS) with INVOS^TM^ for regional cerebral perfusion monitoring, and nociception monitoring with the Analgesia Nociception Index (ANI^®^) to guide analgesic administration to help in managing pain and maintaining stable hemodynamics during the procedure.

Awake fiberoptic intubation was performed to minimize cervical movement under moderate sedation and topical anesthesia with a remifentanil infusion guided by a target-controlled infusion system (TCI) (Minto model). The cervical collar was maintained during intubation. Anesthesia was induced and maintained with remifentanil and propofol infusions guided by TCI, along with intravenous ketamine (0,25 mg/Kg/h) and lidocaine (0,5 mg/Kg/h). There was no use of muscle relaxants. 

Neurophysiological monitoring included SSEPs measured via electrodes placed over the primary somatosensory cortex, with stimulation provided through subdermal needle electrodes at the posterior tibial nerve (medial malleolus) and median nerve (wrist). Warning criteria were defined as a 50% decrease in amplitude or a 10% increase in latency uni- or bilaterally. For transcranial electrical MEPs, stimulation was performed at C3-C4 with corkscrew electrodes, and recordings were made from the first dorsal interosseus (upper limbs) and tibialis anterior and abductor hallucis (lower limbs) muscles. An 80% decrease in amplitude or a 10% increase in latency unilaterally or bilaterally were used as warning criteria.

**Figure 2:**
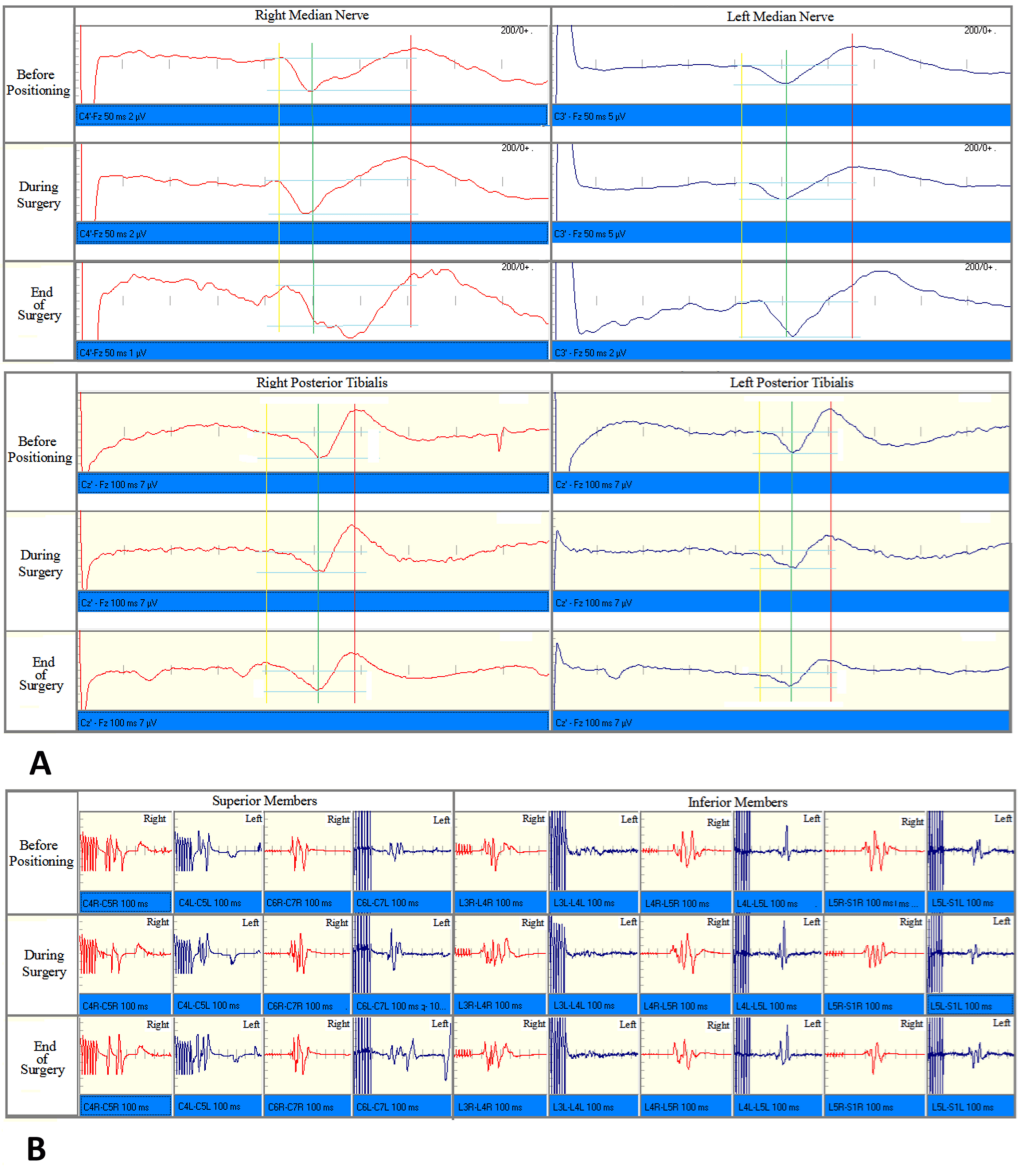
Somatosensory and Motor Evoked Potentials A: Somatosensory Evoked Potentials Before Positioning, During Surgery and End of Surgery B: Motor Evoked Potentials Before Positioning, During Surgery and End of Surgery

After the anesthetic regimen was established and baseline sensory and motor evoked potentials were recorded in the supine position, the patient was positioned for surgery with cervical hyperextension. The baseline responses remained stable during positioning and surgery (Figure 2).

**Figure 3:**
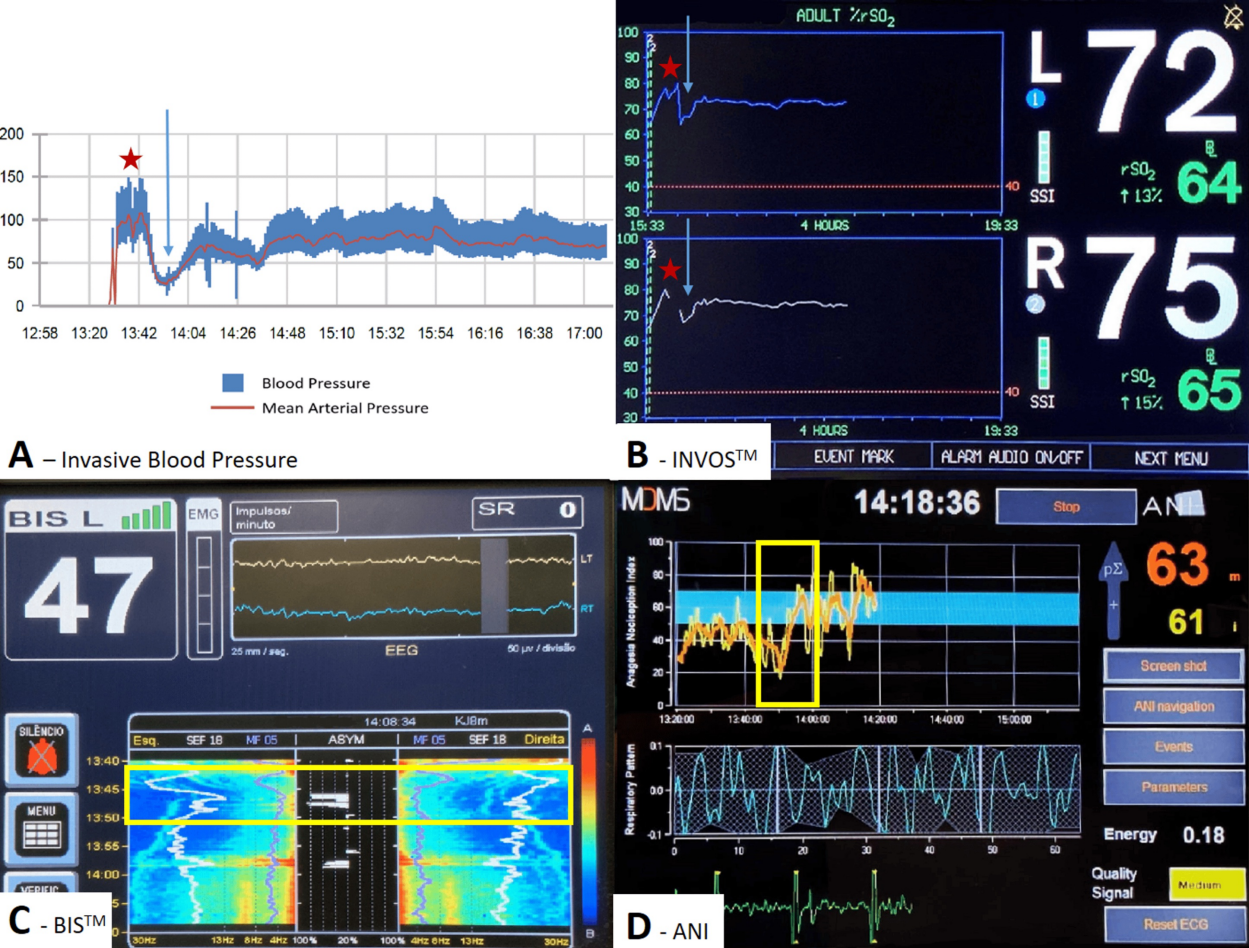
Non-Standard Monitoring with Invasive Blood Pressure Monitoring, rSO2, BIS, and ANI A and B: Blood pressure trends and cerebral oximetry. Star indicates pre-oxygenation baseline; arrow marks the hypotensive episode. C and D: Processed EEG and nociception monitoring. Yellow rectangle highlights the period of hypotension following anesthetic induction.

Following intubation, a hypotensive episode occurred, lasting approximately 10 minutes, with a drop in MAP below 50 mmHg (arrow: Figures 3A, 3B). Concurrently, rSO2 values decreased from 80% (pre-oxygenation level - star: Figure 3B) to 65% (patient's baseline: arrow: Figure 3B). Initially, the hypotension was attributed to an excess of remifentanil due to the absence of surgical stimuli. Consequently, remifentanil effect-site concentration (Ce) was temporarily reduced to help maintain blood pressure. However, this adjustment led to a notable decrease in the ANI (yellow rectangular: Figure 3D), indicating an increased nociceptive response and insufficient analgesia. Additionally, an increase in Spectral Edge Frequency (SEF) was observed (yellow rectangular: Figure 3C). Recognizing the potential for compromised pain control and superficial anesthesia, remifentanil Ce was increased, and 30 mg of ephedrine was administered. No significant alterations in SSEPs and MEPs were observed. This intervention successfully elevated the MAP above 65 mmHg and resulted in a decrease in SEF, though not as dramatically as would be expected with propofol maintenance alone, likely due to the concurrent ketamine infusion (Figure 3).

Following these interventions, hemodynamic stability was maintained throughout the procedure, with systolic blood pressure predominantly above 100 mmHg and MAP above 70 mmHg. Notably, the rSO2 also remained stable, ranging between 70% and 75% throughout the procedure, as there was no significant bleeding or desaturation during the surgery.

The patient emerged from anesthesia without any new neurological deficits. At the three-month follow-up evaluation, the patient demonstrated significant clinical improvement, exhibiting normal sensation and full restoration of muscle strength.

## Discussion

The risk of inducing secondary cervical spinal cord injury during anterior cervical spine surgery is multifactorial, encompassing patient characteristics, intubation techniques, positioning, spinal cord perfusion pressure, and direct surgical interventions.

In patients with cervical myelopathy, the spinal cord is particularly vulnerable to injury, not necessarily from direct trauma, but due to its heightened sensitivity to minor insults that could exacerbate pre-existing conditions [5]. This susceptibility means that seemingly minor factors, such as positioning or slight vascular changes, can result in neurological compromise. Patients with cervical myelopathy are 30% more likely to have significant SSEP and/or MEP amplitude reduction in alerts than those with cervical radiculopathy [5]. In our patient’s case, the combination of cervical myelopathy and trauma presented an elevated risk for secondary spinal cord injury. Recognizing this risk underscores the necessity of heightened vigilance and advanced intraoperative neurophysiological monitoring to prevent the exacerbation of pre-existing lesions and mitigate the risk of secondary injury.

Despite the theoretical risk of cervical spinal cord injury during the induction of general anesthesia and hyperextension of the neck during intubation, actual cases of injury directly attributed to intubation are rare [6]. Regardless of the lack of data associating airway intervention with an increased risk of spinal cord injury, vigilance during intubation remains important. Therefore, awake fiberoptic intubation may be performed in cervical myelopathic patients to prevent hyperextension of the neck, achieve continuous neurological monitoring, and minimize the risk [7].

One of the most critical, yet often overlooked, stages of the procedure that can pose a significant risk to the spinal cord is patient positioning. Unlike intubation, which is brief and generally not monitored with neurophysiological techniques, the positioning of the patient can last for several hours and may involve neck flexion, extension, or traction of the upper extremities. These positions can significantly reduce the diameter of the cervical canal and exert pressure on the spinal cord, especially in patients with pre-existing cervical spine pathology. Despite this risk, positioning is not routinely monitored as closely as other aspects of the surgery [8].

Evidence from previous studies highlights this risk. Morishita et al. identified a significant decrease in SEP amplitude after 20° of cervical extension in patients with cervical spondylotic myelopathy [9]. Similarly, an increase in MEP latency during neck extension has been reported in compressive cervical myelopathy [10]. Additionally, improper arm positioning during surgery can lead to brachial plexus injury, as demonstrated by Jahangiri et al., who reported SEP and MEP deterioration caused by arm positioning during anterior cervical spine surgery [11].

In our case, we took the unique step of initiating SEP and MEP monitoring before patient positioning. Typically, such monitoring is reserved for direct surgical manipulation, but by establishing a reliable baseline of neurophysiological function before any potentially harmful positioning occurred, we could ensure that any neurological alterations were immediately detected and addressed. This proactive approach minimized the risk of injury during this critical phase of the surgery. This case highlights the importance of extending neurophysiological monitoring beyond its conventional use during direct surgical manipulation, particularly during patient positioning, which is a crucial but often overlooked phase of the surgery.

Moreover, vascular compromise during anesthesia, though often underemphasized, can also contribute to secondary spinal cord injury [1]. Controlled hypotension is sometimes employed during surgery to reduce bleeding and improve visibility, but its safety in terms of spinal cord perfusion has not been thoroughly studied [12]. Maintaining normotension, with systolic pressure above 100 mmHg and MAP between 80-85 mmHg, is thought to be the aim of preventing ischemic injury to the spinal cord [1]. When controlled hypotension is necessary, it should be managed carefully to ensure adequate spinal cord perfusion, using global changes in nerve conduction amplitude and latency as warning signs of systemic hypoperfusion [13].

In our case, we implemented a comprehensive monitoring approach to optimize spinal cord perfusion. Beyond neurophysiological monitoring, we utilized continuous invasive arterial blood pressure measurements for precise hemodynamic control. Additionally, we employed rSO2 monitoring as an early detection tool for alterations in systemic organ perfusion, with particular emphasis on spinal cord perfusion [14]. This modality also served to monitor cerebral oxygenation throughout the anesthetic process, ensuring adequate oxygen delivery to the brain and enhancing the overall quality and safety of anesthesia. This multifaceted monitoring strategy enabled real-time assessment and management of the patient's hemodynamic and neurological status. Notably, during a period of hypotension where MAP dropped below 50 mmHg (Figure 3), we observed a corresponding decrease in rSO2 to the patient's baseline. However, as the rSO2 reduction did not exceed 20% from the patient's baseline cerebral oxygenation and no changes were detected in somatosensory evoked potentials SSEPs or MEPs, we concluded that both cerebral oxygenation and spinal cord perfusion were adequately maintained. This comprehensive approach was particularly crucial given the patient's cervical myelopathy and traumatic context. Had changes in SSEPs or MEPs been observed, a more aggressive approach to treating hypotension would have been warranted. This case underscores the importance of multimodal monitoring in guiding anesthetic management and preventing secondary spinal cord injury in high-risk patients undergoing cervical spine surgery.

Surgeons also have the potential to cause direct spinal cord damage during procedures. Intraoperative neurophysiological monitoring, including SSEPs and tceMEPs, provides real-time feedback, allowing for immediate intervention if potential damage is detected [7].

## Conclusions

Multimodal perioperative neurophysiological monitoring, including SSEPs and MEPs, plays an important role in optimizing the surgical and anesthetic management of patients undergoing cervical spine surgery, particularly for those with pre-existing myelopathy. Initiating monitoring before critical stages, such as patient positioning, could help anesthetic teams detect subtle signs of neural compromise early on, potentially allowing for timely technique adjustments. This proactive approach may reduce the risk of iatrogenic spinal cord injury, particularly during positioning - a crucial yet often underappreciated phase of surgery. For patients with myelopathy, where the margin for error is reduced, this strategy may prove beneficial in lowering postoperative neurological complications and enhancing outcomes. Although this report focuses on a single case, it aims to underscore the potential of multimodal monitoring in cervical surgery, thereby encouraging further controlled studies to assess its impact on patient safety and anesthetic practices.
